# Switchable Release of Bone Morphogenetic Protein from Thermoresponsive Poly(NIPAM-*co*-DMAEMA)/Cellulose Sulfate Particle Coatings

**DOI:** 10.3390/polym10121314

**Published:** 2018-11-27

**Authors:** Martin Müller, Birgit Urban, Berthold Reis, Xiaoqian Yu, Anna Luise Grab, Elisabetta Ada Cavalcanti-Adam, Dirk Kuckling

**Affiliations:** 1 Leibniz-Institut für Polymerforschung Dresden e.V., Abteilung Polyelektrolyte und Dispersionen, Hohe Straße 6, 01069 Dresden, Germany; urban@ipfdd.de (B.U.); reis@ipfdd.de (B.R.); 2 Technische Universität Dresden, Fachrichtung für Chemie und Lebensmittelchemie, 01062 Dresden, Germany; 3 Universität Paderborn, Department Chemie, Organische und Makromolekulare Chemie, Warburger Str. 100, 33098 Paderborn, Germany; yu.xiaoqian1987@hotmail.com (X.Y.); dirk.kuckling@uni-paderborn.de (D.K.); 4 Medizinische Klinik V, Universitätsklinikum Heidelberg, INF 350, 69120 Heidelberg, Germany; agrab@ix.urz.uni-heidelberg.de; 5 Institute of Physical Chemistry, Department of Biophysical Chemistry, Heidelberg University, INF 253, 69120 Heidelberg, Germany; elisabetta.cavalcanti-adam@mpimf-heidelberg.mpg.de; 6 Max Planck Institute for Medical Research, Department of Cellular Biophysics and Central Scientific Facility “Cell Biology”, Jahnstr. 29, 69120 Heidelberg, Germany

**Keywords:** bone healing, protein delivery, polyelectrolyte complex, thermoresponsive polymers, bone morphogenetic protein 2

## Abstract

Thermoresponsive coatings of poly(*N*-isopropylacrylamide-*co*-DMAEMA)/cellulose sulfate (PNIPAM-DMAEMA/CS) complexes are reported eluting bone-morphogenetic-protein-2 (BMP-2) on demand relevant for implant assisted local bone healing. PNIPAM-DMAEMA/CS dispersions contained colloid particles with hydrodynamic radii R_H_ = 170–288 nm at T = 25 °C shrinking to R_H_ = 74–103 nm at T = 60 °C. Obviously, PNIPAM-DMAEMA/CS undergoes volume phase transition (VPT) analogously to pure PNIPAM, when critical VPT temperature (VPTT) is exceeded. Temperature dependent turbidity measurements revealed broad VPT and VPTT 47 °C for PNIPAM-DMAEMA/CS colloid dispersions at pH = 7.0. FTIR spectroscopy on thermoresponsive PNIPAM-DMAEMA/CS particle coatings at germanium model substrates under HEPES buffer indicated both wet-adhesiveness and VPT behavior based on diagnostic band intensity increases with temperature. From respective temperature courses empirical VPTT ≈ 42 °C for PNIPAM-DMAEMA/CS coatings at pH = 7.0 were found, which were comparable to VPTT found for respective dispersions. Finally, the PNIPAM-DMAEMA/CS coatings were loaded with BMP-2 and model protein papain (PAP). Time dependent FTIR spectroscopic measurements showed, that for T = 37 °C there was a relative protein release of ≈30% for PAP and ≈10% for BMP-2 after 24 h, which did not increase further. Heating to T = 42 °C for PAP and to 47 °C for BMP-2 further secondary protein release of ≈20% after 24 h was found, respectively, interesting for clinical applications. BMP-2 eluted even at 47 °C was found to be still biologically active.

## 1. Introduction

Recently, we reported on a thermoresponsive polyelectrolyte complex (PEC) based drug delivery coating consisting of the random copolymer of *N*-isopropylacrylamide and acrylic acid (PNIPAM-AA), which was complexed with cationic ethylenediamine modified cellulose (EDAC) [1]. Pure PNIPAM and systems like copolyelectrolytes of NIPAM feature a volume phase transition (VPT) on the macroscopic level due to coil/globule transition and change of the hydration state of NIPAM segments on the microscopic level, when a certain volume phase transition temperature (VPTT) is exceeded. For pure PNIPAM the VPTT is around 33 °C [2,3]. Into the above mentioned EDAC/PNIPAM-AA coatings, the low molecular anionic drug zoledronate (ZOL) for bone healing was loaded and an increased ZOL elution with increased temperature was demonstrated [1]. This finding is relevant for the functionalization of bone substituting materials (BSM) with drug delivery systems operating on-demand, i.e., induced by an external stimulus, which is relevant for implant assisted local bone healing. Besides temperature, such a stimulus can also be applied for example chemically, electrically, acoustically or magnetically as it was reviewed [4].

Herein we would like to go further and extend this development from charged low molecular drugs to higher molecular functional proteins used for bone healing. Among those proteins certain growth factors like bone morphogenetic proteins (BMP) have attracted considerable interest [5]. Since around 1970 BMPs, which belong to the TGF-β class, have been identified as essential molecules for the de-novo formation of animal bones [6] and as the strongest known osteoinductive factor. They regulate cell proliferation, differentiation, motility and survival from the embryonic phase until the adult phase and especially promote differentiation of myoblasts into osteoblasts and their maturation. They are also used in tissue engineering approaches procedures such as spine surgery. The molecular structure of BMPs like BMP-2 is known from protein crystallography [7]. BMP-2 has a molecular weight around 30.000 g/mol, possesses a high content of α-helix/β-sheet and an isoelectric point of IEP = 8.5, classifying it as a basic cationic protein at the physiological pH = 7.0. This cationic property is important for the integration process of the protein drug used in our approach. While in our former study the low molecular anionic drug ZOL was electrostatically bound to PEC particles with a cationic net charge, herein higher molecular proteins with a cationic net charge shall be bound to PEC coatings, which have an anionic net charge.

In this report instead of the established thermoresponsive PEC system EDAC/PNIPAM-AA [1] the system of poly(*N*-isopropylacrylamide-*co*-dimethylaminoethylmethacrylate)/cellulose sulfate (PNIPAM-DMAEMA/CS) shall be introduced. Three topics are focused in this report, which are colloidal properties and VPT behavior of PNIPAM-DMAEMA/CS in the bulk dispersion (i), interfacial properties and VPT behavior of the PNIPAM-DMAEMA/CS coating (ii) and thermoinducable release of model protein papain and biomedically relevant BMP-2 out of this coating (iii).

## 2. Materials and Methods

### 2.1. Materials and Reagents

**Figure 1:**
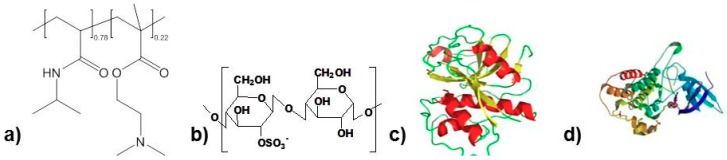
Formulae of PNIPAM-DMAEMA (**a**), CS (**b**), BMP-2 (**c**) and papain (**d**).

The thermoresponsive cationic copolyelectrolyte poly(*N*-isopropylacrylamide-*co*-dimethylaminoethyl-methacrylate) (PNIPAM-DMAEMA, random copolymer, M_n_ = 7.200 g/mol, NIPAM mole percent: 78%, DMAEMA mole percent: 22%, Figure 1a) was prepared as follows: 2-(dimethylamino)ethyl methacrylate (DMAEMA, Aldrich, Darmstadt, Germany) (0.795 g, 5.1 mmol), *N*-isopropylacrylamide (NIPAM, TCI, Eschborn, Germany) (1.679 g, 14.8 mmol), 4-cyano-4-(((dodecylthio)carbon-thioyl)thio)pentanoic acid (CDPA, Aldrich, Darmstadt, Germany) (30 mg, 0.074 mmol), 4,4′-azobis(4-cyanovaleric acid) (ACVA, Aldrich, Darmstadt, Germany) (2 mg, 0.007 mmol) and 1,4-dioxane (5 mL) were placed in a Schlenk tube, which was equipped with magnetic stirrer and septum. The reaction mixture was purged with argon for 20 min and the Schlenk tube was immersed into an oil bath at 70 °C to start the copolymerization. After 24 h the polymerization was stopped by freezing the sample in liquid nitrogen. 1,4-dioxane was evaporated in vacuo and the residue was dissolved in THF. The polymer was purified by precipitations in cold diethyl ether and dried with fine vacuo at room temperature. The conversion and polydispersity of PNIPAM-DMAEMA copolymer were determined from ^1^H-NMR and SEC analysis. The polyanion cellulose sulfate (CS, degree of substitution d_S_ = 0.5, M_W_ = 100.000 g/mol, Euroferm, Erlangen, Germany, Figure 1b) was used for complexation. PNIPAM-DMAEMA and CS were diluted to 0.01 M solutions related to their repeating units, respectively. For PNIPAM-DMAEMA the weighted (78%/22%) average (123 g/mol) of the molecular weights of NIPAM (113 g/mol) and DMAEMA (158 g/mol), respectively, was taken into account (see Table 1). The pH value of the polyelectrolyte solutions was adjusted by addition of HCl (0.1 M) or NaOH (0.1 M). For reference measurements pure PNIPAM (M_W_ = 100.000 g/mol, Polysciences Europe, Eppelheim, Germany) was used. The proteins papain (PAP, Sigma, Darmstadt, Germany, Figure 1c), which has molecular weight of M_n_ ≈ 24.000 g/mol and isoelectrical point (IEP) = 8.75 [8], and bone morphogenetic protein-2 (BMP-2, ProSpec GmBH, Heidelberg, Germany, Figure 1d), which has M_n_ ≈ 30.000 g/mol and IEP ≈ 8.5 [7] were used in this report. Diluted protein stock solution of 0.001 M was prepared and adjusted to pH = 7.0 by dropwise addition of 0.1 M HCl.

### 2.2. Polyelectrolyte Complex (PEC) Preparation

Cationic PNIPAM-DMAEMA and anionic CS were complexed for the two molar mixing ratios n−/n+ = 0.9 (denoted PEC-0.9) and n−/n+ = 1.2 (PEC-1.2) at either pH = 7.0, respectively. n− and n+ denote molar concentrations of negative and positive charges at PEL, respectively, and are experimentally measured by colloid titration (using particle charge detector, see below). For PEC-0.9 samples 0.01 mol/L PNIPAM-DMAEMA solution (pH = 7.0) was presented and 0.01 mol/L CS solution (pH = 7.0) was added, while for PEC-1.2 samples the 0.01 mol/L CS solution (pH = 7.0) was presented and the 0.01 mol/L PNIPAM-DMAEMA solution (pH = 7.0) was added. Slightly turbid PEC dispersions were obtained for both systems at T = 25 °C (Thermostat, Haake GmbH, Vreden, Germany), while more turbid (milky) ones were obtained at T = 60 °C. PNIPAM-DMAEMA/CS dispersions were brought to pH = 4.0 and 9.0 by adding 0.1 M HCl or 0.1 M NaOH to the PEC dispersion originally prepared at pH = 7.0.

### 2.3. PEC Coating Preparation

PNIPAM-DMAEMA/CS coatings were prepared by casting typically 50 μL of the given PEC dispersion onto germanium (Ge) model substrates, which were dried at T = 50 °C (oven) for 15 min resulting in a wet coverage of A = 3.0 ± 0.2 cm^2^.

### 2.4. Protein Loading and Release

Protein (PAP, BMP-2) loading of initially dry PEC coatings was achieved by immersing the PEC coated Ge substrates into 0.001 M protein solution at pH = 7.0 for at least 12 h. Afterwards the wet PEC coated Ge substrates loaded by protein were withdrawn, put on paper tissue and immediately the remaining liquid was carefully removed by N_2_ flow. When this was done with the uncoated Ge substrates, no measurable dry traces after the analogous removal procedure were observed by FTIR spectroscopy (see below). Protein release was measured similarly by FTIR spectroscopy, whereby the protein loaded PEC covered Ge substrates were dipped into HEPES buffer solution at pH = 7.0, withdrawn from the release medium after defined time periods and treated similarly before FTIR measurement (see below). After that the protein loaded PEC covered Ge substrates were again dipped in HEPES buffer at pH = 7.0.

### 2.5. Colloid Titration

Colloid titration was used to determine molar charge concentrations (n−, n+) in PNIPAM-DMAEMA and CS solutions, which are not per se identical to molar concentrations of the repeating units (c_PC_, c_PA_). The measurements where conducted with the particle charge detector (Mütek PCD-04 and Mütek PCD-T3, BTG Instruments, Herrsching, Germany). Thereby a given PEL solution is titrated by an oppositely charged low molecular PEL solution of either poly(diallyldimethylammonium chloride) for negative charges or poly(ethylenesulfonate) for positive charges until the value of the measured zeta-potential [mV] reaches zero. Typically, PEL solutions with volumes of 10 mL and concentrations (c_PEL_) of 0.0001 mol/L (based on molecular weight of repeating units) are titrated by adding volumes of titrator solutions of 0.001 mol/L (V_TIT_).

The volume ratio F = V_TIT_/V_PEL_ defines the so called charge factor, from which n− or n+ can be calculated according to n− = F c_PA_ and n+ = F c_PC_, where c_PC_ and c_PA_ are the concentrations of polycation (PC) and polyanion (PA) related to repeating units. Table 1 summarizes values of charge factors for the used PEL, which are further used to obtain the mixing ratios n−/n+ = 0.9 and n−/n+ = 1.2 in this study.

### 2.6. Turbidity

Temperature dependent turbidity measurements on PNIPAM-DMAEMA solutions and PNIPAM-DMAEMA/CS dispersions were performed at a UV/VIS spectrometer (JASCO 630, JASCO GmbH, Eschborn, Germany) recording the absorbance at 450 nm for temperatures in the range 25 to 70 °C using a thermostatable cuvette holder (cuvette pathway: 2 mm). Absolute turbidity values were measured after 30 min equilibration time at each temperature. Normalized turbidity values (values between 0 and 1) were obtained by ratioing given absolute turbidity values by the last measured one with highest absolute turbidity.

### 2.7. Dynamic Light Scattering (DLS)

Hydrodynamic radii (R_H_) of PNIPAM-DMAEMA/CS PEC dispersions were determined using DLS (Jianke Portable Particle Sizer, Jianke Instruments Co., Ltd., Wuhu, China) at a scattering angle of 89.3°. PEC dispersions (2 mL) were measured in 10 mm cuvettes with circular bottom recording the autocorrelation function for 180 s. The R_H_ value was calculated from the translational diffusion coefficient D using Stokes-Einstein equation R_H_ = k_B_T/(D 6 π η) with Boltzmann constant k_B_, temperature T (25 or 60 °C) and viscosity η. Intensity weighted size distributions were considered. Error values of R_H_ were related to the standard deviation among 3–5 equally prepared but individual PEC dispersion samples. For the computation of DLS parameters ALV-5000/E/EPP-Software (ALV GmbH, Langen, Germany) was used applying the tool “Simple Fit”.

### 2.8. Fourier Transform Infrared (FTIR) spectroscopy

FTIR spectrometer (Tensor 27, BRUKER-Optics GmbH, Ettlingen, Germany) equipped with globar source and either deuterated triglycine sulfate (DTGS) or mercurium-cadmium-telluride (MCT) detector was used in the mid-IR range. FTIR spectra were recorded in the wavenumber range between 4000–400 cm^−1^ at 2 cm^−1^ spectral resolution and 50 scans were coadded. Both modes ex-situ transmission (TRANS) and in-situ attenuated total reflection (ATR) were used.

TRANS-FTIR was applied at germanium (Ge) internal reflection elements (IRE), which were fixed in constructed sample holders (M. Ulrich, M.M., IPF Dresden). The IR beam transmitted the Ge IRE at minimum focal area (<5 mm) in the FTIR sample chamber. IR absorbance spectra (A(ν)) were recorded from polymer coated Ge IREs as the sample (I_S_(ν)) using clean (plasma cleaner, Harrick, Ossining, NY, USA) uncoated Ge IREs as the reference (I_R_(ν)) and processed according to A(ν) = −log I_S_(ν)/I_R_(ν). PEC films were typically measured after drying under a gentle N_2_ gas stream (dry state).

ATR-FTIR was applied at the in-situ-ATR liquid cell (M.M., IPF Dresden) located in a commercial pseudo-double-beam-ATR device (OPTISPEC, Neerach, Switzerland), which was operated according to the single-beam-sample-reference (SBSR) concept of Fringeli [10]. The in-situ-ATR liquid cell consists of two sample compartments (front and back) on the upper half and two reference compartments (front and back) on the lower half sealing a trapezoidal germanium IRE (50 × 20 × 2 mm^3^) allowing an incident and leaving angle of 45°. Sample (I_S_) and reference (I_R_) halves of the Ge IRE are alternately shuttled into the IR beam by lift and absorbance spectra are computed according to A = −log I_S_/I_R_. Typically, dry PEC films or liquid PEC dispersions are on upper and bare surface or aqueous solvent on lower half of Ge.

### 2.9. Wet Adhesion of PNIPAM-DMAEMA/CS Films

TRANS-FTIR spectra of PEC coatings prepared as described above were recorded in the dry state before and after rinsing in aqueous media (HEPES, pH = 5.0, pH = 9.0 and 0.1 M NaCl) for 1 h at T = 25 and 60 °C. Integrals of diagnostic IR bands for each of the PEL components (PNIPAM-DMAEMA: Amide II; CS: ν(C–O) of saccharide groups) were determined for the IR spectrum of the PEC coating before (A_BEFORE_) and that after rinsing (A_AFTER_) in aqueous medium. The percentage ratio W = A_AFTER_/A_BEFORE_ * 100% was calculated as a measure for PEC film wet-adhesive stability. At least three independent measurements were made for each parameter combination (NaCl, pH, T).

### 2.10. Volume Phase Transition of PNIPAM-DMAEMA/CS Films

In-situ ATR-FTIR spectra of the PEC coating in contact to HEPES buffer (pH = 7.0), water at pH = 5.0 and pH = 9.0 and to sodium chloride (0.1 M NaCl) solution were recorded at temperatures in the range T = 20–75 °C in a special thermostatable cell (IPF construction). As references intensity spectra (I_R_) of these aqueous media (HEPES, pH = 5.0, pH = 9.0 and 0.1 M NaCl) at the respective temperature (20–75 °C) were used. Otherwise baseline shifts would be obtained. The integral of the Amide II band (1590–1510 cm^−1^) was determined and plotted versus temperature.

### 2.11. Protein Content in PNIPAM-DMAEMA/CS Films

The actual relative protein content L in the PNIPAM-DMAEMA/CS film was determined by TRANS-FTIR in the dry state as it was described earlier for a low molecular drug [1]. Herein the integral of Amide II band (mixture of N-H bending and C–N stretching vibration) of both protein and PNIPAM-DMAEMA located at around 1550 cm^−1^ (A_1550_) was ratioed with that of the ν(C–O) band (C–O–C, C–O–H group vibration) of CS at 1065 cm^−1^ (A_1065_) according to P = A_1550_/A_1065_. Calculating P for the bare PNIPAM-DMAEMA/CS film before loading the reference ratio P_R_ (no loading) is defined. Calculating P for the film after complete (maximum) loading of protein for 12h the initial sample ratio P_S0_ and for the actual films (loadings) at given times t the actual sample ratios P_S_ (actual loading) were determined. The following Equation (1) was used for calculation of actual relative protein contents in percentage:

L = (P_S_ − P_R_) (P_S0_ − P_R_) × 100% (1)

### 2.12. Scanning Force Microscopy (SFM)

SFM on PEC films was performed using Ultramicroscope Nanostation II (Bruker Nano GmbH (Karlsruhe, Germany). Silicon tips (10 nm apices) from Nanosensors (Darmstadt, Germany) were used. Images from dry state samples were recorded in “non-contact mode” in topography, error and phase mode, where scanning parameters were optimized under the condition of minimum amplitudes for the error mode. Microscopic data was further processed by SISCAN-Pro software (BRUKER Nano GmbH, Karlsruhe, Germany).

### 2.13. Spectroscopic Reflectometry (SR)

Thicknesses of dry PECNP coatings at Ge substrates were determined applying Spectroscopic Reflectometry. The thin film measurement system (MProbeVis-MSP) of Semiconsoft Inc. (Southborough, MA, USA) including a light source of tungsten-halogen (5 W), fibre optics probe and F4 Vis spectrometer with Si detector in the wavelength range 400–1100 nm was used. Due to inhomogeneity of the sample films thickness values d were crucially dependent on the location of the measurements spot. Therefore, three independent samples of PNIPAM-DMAEMA/CS films were measured at nine different positions, (three rows, three columns) respectively. All 27 determined thicknesses (three samples, 9 positions) were averaged, given herein as the thickness value d, and the standard deviation determined.

### 2.14. Cell Culture and Western Blotting

Biological activity of BMP-2 released from PNIPAM-DMAEMA/CS films within 24 h at 37 °C (sample A) and 47 °C (sample B) was investigated by activation of BMP-mediated SMAD-dependent pathway in C2C12 myoblasts. Bioactive BMP-2 triggers Smad 1/5 phosphorylation and was determined using western blotting. In brief, mouse C2C12 myoblasts (ATCC CRL-1772) were cultured in Dulbecco’s modified Eagle’s medium (DMEM, 41966-029, Gibco LifeTechnologies, Carlsbad, CA, USA) containing 10% (*v*/*v*) fetal bovine serum (FBS, S0115m, Biochrom AG, Berlin, Germany) and 1% (*v*/*v*) penicillin/streptomycin (15140, Gibco). Cells were seeded overnight in 6 well plates with a density of 10,000 cells/cm^2^. 1 mL of the medium was removed and replaced by 600 µL sample A or sample B. For negative control (sample C) 600 µL buffer without BMP-2 was used. After 1h cells were washed with PBS and lysed in 2X Laemmli Buffer (consisting of 4% SDS, 20% Glycerol, 120 mM Tris–HCl, pH = 6.8, 200 mM DTT, 0.02% Bromphenolblue), frozen for at least 4 h and boiled at 97 °C for 3 min. The total proteins homogenates were loaded onto SDS-PAGE (NuPAGE 4–12% Bis–TrisGel) and separated according to manufacturer’s manual using MES Running Buffer (Novex, LifeTechnologies, Carlsbad, CA, USA). Proteins were blotted into nitrocellulose membrane (GEHealthcare, Little Chalfont, UK) in NuPAGE Transfer Buffer (Novex, LifeTechnologies, Carlsbad, CA, USA). After transfer the membrane was blocked against nonspecific adsorption with 5% BSA (Sigma-Aldrich, St. Louis, MO, USA) dissolved in TBST buffer (50 nM Tris-buffered saline, pH = 8.0, 150 mM NaCl, 0.1% Tween-20) for at least 1 h at RT and incubated with rabbit anti pSMAD 1/5 (#9516, CellSignaling, Danvers, MA, USA) 1:1000 in 1% BSA in TBST, washed with TBST and incubated for 1 h with HRP-conjugated secondary antibody goat anti-rabbit 1:5000 in 1% BSA in TBST. For loading control the membrane was incubated with mouse antiβ-actin (A1978, Sigma-Aldrich, St. Louis, MO, USA) for 1 h and with HRP-conjugated secondary antibody goat anti-mouse (Santa Cruz Biotechnology, Heidelberg, Germany) 1:5000 in 1% BSA and imaged with Amsterham Imager 600 and visualized using imageJ (version 1.51).

## 3. Results and Discussion

In the following structure and properties of thermoresponsive poly(*N*-isopropylacrylamide-*co*-dimethylaminoethylmethacrylate)/cellulose sulfate (PNIPAM-DMAEMA/CS) complexes in dispersions and coatings as well as their usage for the switchable release of model proteins and bone morphogenetic protein 2 (BMP-2) are reported. At first bulk properties of PNIPAM-DMAEMA/CS dispersions for the two molar mixing ratios n−/n+ = 0.9 and n−/n+ = 1.2 in dependence of temperature are presented (Section 3.1). Secondly, interfacial properties of PNIPAM-DMAEMA/CS coatings attached to a germanium (Ge) model surface at similar molar mixing ratios and temperature range in contact to aqueous media are reported (Section 3.2). Finally, drug delivery properties of PNIPAM-DMAEMA/CS coatings at Ge model surface with respect to the model protein papain and the therapeutic bone morphogenetic protein (BMP-2) as a function of temperature are presented (Section 3.3).

### 3.1. Colloid and Thermal Properties of PNIPAM-DMAEMA/CS Dispersions

#### 3.1.1. Turbidity

**Figure 2:**
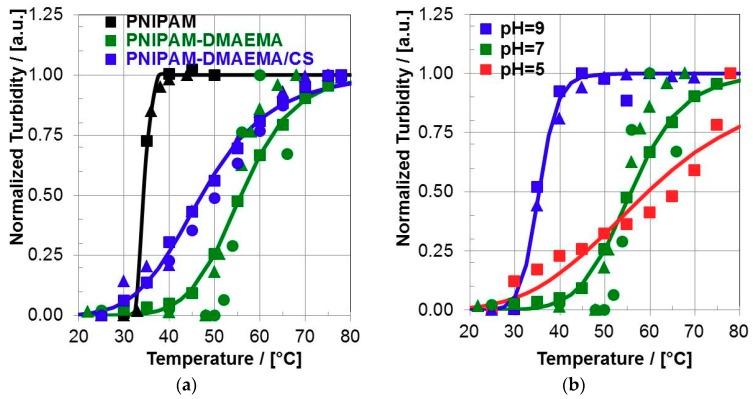
(**a**) Turbidity of PNIPAM (black) and PNIPAM-DMAEMA (green) solutions (0.02 M) compared to PNIPAM-DMAEMA/CS (0.01 M) dispersions (blue) for n−/n+ = 1.2 at pH = 7.0 as a function of temperature. (Cubes, triangles and circles denote different independent measurements, full lines correspond to fitted curves according to Equation (2)); (**b**) Turbidity of PNIPAM-DMAEMA solutions (0.02 M) at pH = 5.0 (red), pH = 7.0 (green) and pH = 9.0 (blue) as a function of temperature. (Cubes, triangles and circles denote different independent measurements, full lines correspond to fitted curves according to Equation (2)).

At first turbidity of PNIPAM-DMAEMA/CS dispersions was measured in dependence of temperature. In the Figure 2a respective absorbance values at 450 nm of PNIPAM-DMAEMA/CS dispersions together with PNIPAM-DMAEMA and PNIPAM solutions at pH = 7.0 are plotted versus temperature.

As it is well known PNIPAM solutions reveal sharp thermal volume phase transitions (VPT) with volume phase transition temperatures (VPTT), usually denoted as lower critical solution temperatures LCST, of around 33 °C [2]. Generally, in this work values for VPTT were determined by fitting the data with a logistic function (Equation (2)), which can be used to describe cooperative phase transition processes:

Q(T) = Q_MAX_ + (Q_MIN_ − Q_MAX_)/(1 + (T/VPTT)^P^) (2)

Thereby beside VPTT the parameters Q_MIN_ and Q_MAX_ denote the onset and the upset of Q and the exponent P denotes the cooperativity factor, which is higher the steeper (less broad) is the phase transition. The found VPTT and P values are given in the Table 2.

First of all, for PNIPAM solutions a sharp most pronounced VPT was found characterized by a VPTT = 34.1 °C and a high cooperativity value of P = 34.5. Whereas, solutions of PNIPAM-DMAEMA show a significant but moderately pronounced VPT with a shifted VPTT = 55.8 °C and a moderate cooperativity value of P = 9.7. Finally, PNIPAM-DMAEMA/CS complex dispersions show broader and less pronounced VPT with a VPTT = 47.1 °C and a low cooperativity value of P = 6.2.

Furthermore, the influence of pH on the VPT of P(NIPAM-DMAEMA) solutions was studied, which is given in the Figure 2b. Thereby, at pH = 9.0 a similar transition temperature of VPTT = 35.3 °C with a moderate P = 17.2 compared to pure PNIPAM was found, while at pH = 5.0 values of VPTT = 59.6 °C and P = 4.2 were found. Obviously at pH = 9.0, DMAEMA segments are uncharged or lower charged, so that no additional hydrophilic groups are present, which could bind water and thus disfavor VPT. Therefore, the VPTT value is similar to the LCST value of pure PNIPAM. In contrast, at pH = 5.0 DMAEMA segments are charged and hydrophilic and bind strongly water and thus the VPT is hindered and shifts VPTT to values far beyond the LCST of pure PNIPAM. Molecular explanations include the well-known coil/globule transition of NIPAM segments due to the loss of hydration and external hydrogen bonding by water molecules upon temperature increase [2], which is hindered when charged hydrophilic comonomer units like DMAEMA prevent the dehydration to a certain extent. Additionally, self-repulsion by the like charges of the DMAEMA units contribute to the higher LCST value of PNIPAM-DMAEMA. Thus VPTT values increase for both pure and complexed PNIPAM-DMAEMA system as well as the transition gets broader. However, such broad VPT and VPTT values exceeding the physiological temperature of T_PHYS_ = 37 °C are still relevant for applications in biomedicine, since structural changes in the PNIPAM-DMAEMA phase already start before and after reaching T_PHYS_ = 37 °C. Therefore, a temperature scaled influence on the drug delivery properties (drug retention and release) might be expected, which is treated further below.

#### 3.1.2. Particle Size

Furthermore, temperature dependent DLS measurements on PNIPAM-DMAEMA/CS dispersions (0.01 M) for the mixing ratios n−/n+ = 0.9 and 1.2 at pH = 4.0 and 7.0 were performed. In the Table 3 hydrodynamic radii (RH) of PNIPAM-DMAEMA/CS complex dispersions at T = 25 and 60 °C are given.

Generally, for T = 25 °C both PNIPAM-DMAEMA/CS-0.9 and PNIPAM-DMAEMA/CS-1.2 dispersions at pH = 7.0 showed similar values of R_H_ = 171 and 170 nm, respectively. Whereas PNIPAM-DMAEMA/CS-1.2 dispersions at pH = 4.0 showed significantly higher values of R_H_ = 288 nm, which can be explained by the increase of charge density within PNIPAM-DMAEMA at pH = 4.0 compared to pH = 7.0 causing a more stretched conformation and thus PEC particle increase. PNIPAM-DMAEMA/CS-0.9 dispersions at pH = 4.0 were colloidally instable and resulted in flocculation. Increasing the temperature from T = 25 to 60 °C all stable PNIPAM-DMAEMA/CS samples at pH = 7.0 and 4.0 revealed a significant decrease of particle size to values of R_H_ = 103–74 nm, which indicates shrinkages by factors of 1.7–3.9 at T = 60 °C with respect to the swollen state at 25 °C. This can be explained by the volume phase transition of the PNIPAM moieties causing deswelling of the whole PEC particle, which is in line with the known shrinkage of pure PNIPAM [11] as well as pure uncomplexed PNIPAM-AA [12] due to the coil/globule transition.

### 3.2. Interfacial and Thermal Properties of PNIPAM-DMAEMA/CS Coatings

#### 3.2.1. Wet-Adhesiveness of the Coating

**Figure 3:**
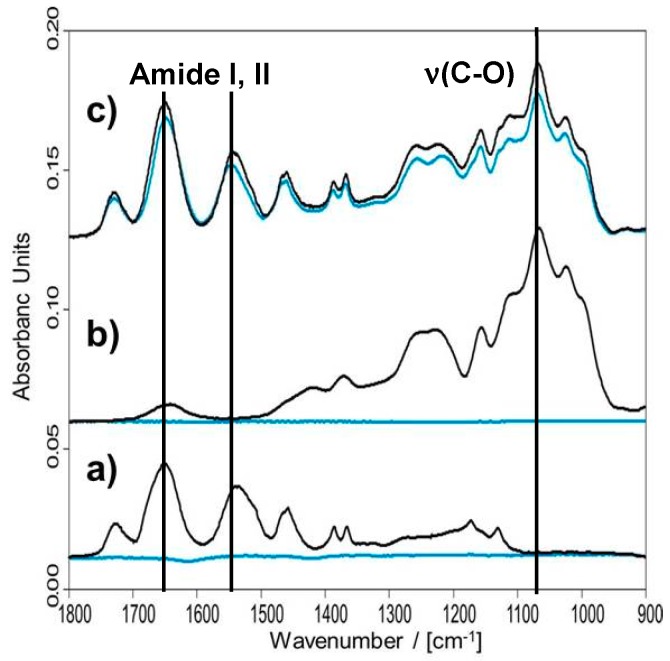
Transmission FTIR spectra of casted films of PNIPAM-DMAEMA (**a**), CS (**b**) and PNIPAM-DMAEMA/CS-1.2 (**c**) at Ge/GeO_x_H_y_ in the initial dry state (black) and the dry state after rinsing in HEPES buffer at pH = 7.0 (blue), respectively.

First of all, the wet-adhesiveness of the PNIPAM-DMAEMA/CS coatings was studied, which is important for further measurements, especially those related to thermal characterization (see Section 3.2.3) and drug delivery (see Section 3.3). Wet-adhesiveness was checked by FTIR spectroscopy in the dry state. In the Figure 3 related FTIR spectra on casted films of the single components PNIPAM-DMAEMA, CS and the complex PNIPAM-DMAEMA/CS-1.2 in the initial dry state before (black) and the dry state after rinsing (blue) in HEPES buffer are shown.

Generally, the FTIR spectra of PNIPAM-DMAEMA/CS are dominated by the intense Amide I (80% (C=O) stretching vibration) and Amide II (60% (N–H) bending vibration) bands of the NIPAM repeating units at around 1630 and 1550 cm^−1^, respectively and the ν(C=O) band of the ester group of the DMAEMA repeating units at around 1730 cm^−1^. Furthermore, at around 1065 cm^−1^ the maximum of a strong composed IR band mainly due to the ν(C–O) stretching band of C–O–C and C–O–H groups of the polysaccharide CS is present. The spectra of the (binary) PNIPAM-DMAEMA/CS-1.2 coatings before rinsing did not decrease substantially in their intensities compared to after rinsing in HEPES buffer (pH = 7.0), while spectra intensity of the films of the single components decreased significantly. Slight deviations from 100% by some 10% are due to slight local losses of the PNIPAM-DMAEMA coating or different degrees of swelling in the sample before and after rinsing. Similar effects were recently reported at comparable thermoresponsive coatings [1], which also showed significant rinse stabilities against buffer solutions. Hence, obviously PNIPAM-DMAEMA can be surface attached, when it is complexed with oppositely charged polyelectrolytes like CS and deposited by drying resulting in rinse-stable thermoresponsive coatings, while casted films of the pure uncomplexed PNIPAM-DMAEMA and pure uncomplexed CS component dissolved readily in HEPES buffer solution.

To quantify empirically the wet-adhesiveness (W) intensity ratios of band areas of the Amide II band at around 1550 cm^−1^, which is diagnostic for PNIPAM-DMAEMA compound, and of the ν(C–O) band at around 1065 cm^−1^, which is diagnostic for CS compound, after (A_AFTER_) and before rinsing (A_BEFORE_) with water, respectively, were calculated according to W = A_AFTER_/A_BEFORE_ × 100%. These values are listed in Table 4.

Generally, wet-adhesiveness values of PEC coating samples were in the range W = 66–93% for T = 25 and 60 °C. Hence, most of the PEC coating remained stable, but in some cases around 7–34% was lost. In detail the PNIPAM-DMAEMA compound was lost to a minor and the CS compound was lost to a larger extent. As an explanation CS was in a slight excess over the PNIPAM-DMAEMA component and not electrostatically loosely bound parts could be rinsed off easier as we pointed out for another PEC system therein [13]. Furthermore, pH = 7.0 was optimum for wet-adhesiveness, while at pH = 9.0 largest loss of PEC coating was observed. Consequently, for further loading and releasing studies as well as cell experiments PNIPAM-DMAEMA/CS films must be thoroughly rinsed after their deposition, so that loosely bound material is eliminated. The temperature increase from T = 25 to 60 °C revealed no significant effect on the wet-adhesiveness.

#### 3.2.2. Morphology and Thickness of the Coating

**Figure 4:**
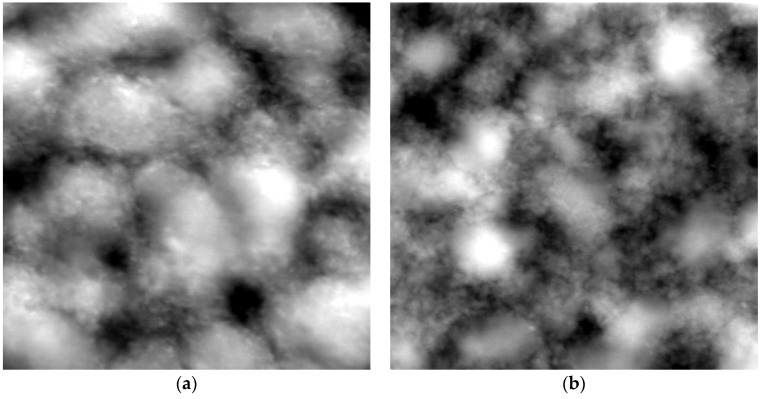
SFM images (4 × 4 μm^2^, topography) of PNIPAM-DMAEMA/CS-1.2 coatings deposited at T = 50 °C and pH = 4 onto Si substrate. (**a**) Initial state before rinsing with pure water; (**b**) state after rinsing with pure water.

In the Figure 4 SFM images of PNIPAM-DMAEMA/CS-1.2 coatings deposited from PECNP dispersions at pH = 4.0 and T = 50 °C in the initial state (Figure 4a) and after rinsing (Figure 4b) are shown.

Generally, granular morphologies were obtained originating from single and partly fused PECNP. Particular objects in Figure 4a suggest overall sizes in the submicron to micron range. Larger sizes compared to those from DLS measurements (Table 3) were obtained, which can be explained by spreading of the PECNP on the Si substrates. Interestingly, the micron sized PECNP seem to be further composed of smaller particles in the size range of around 50–100 nm. This observation is in line to earlier experimental observations and simulations on PECNP composed of the two synthetic PELs PDADMAC and PSS [14,15]. Therein we found evidence, that the PEL complexation process includes a rapid part, where small molecular complexes (primary PEC particles, 1–10 nm) of few polycation and polyanion molecules are formed by ion pairing (long range electrostatic interactions) and a slower part, where these primary PEC further aggregate to secondary PEC particles (100–1000 nm) by short range non-electrostatic interactions, which reach colloidal stability at a certain particle size due to mutual electrosteric repulsion. As it is shown in the Figure 4b rinsing of the PECNP film results neither in detachment nor in significant morphology change due to the adhesiveness evidenced in Table 4.

Moreover, the thickness d of PNIPAM-DMAEMA/CS-1.2 coatings at T = 25 °C casted from PEC dispersions at pH = 7.0 before and after rinsing in H_2_O (pH = 7.0) was determined by spectroscopic reflectometry. Due to inhomogeneity of the PEC sample films broad range of d values were found, since d was critically dependent on the location of the measurements spot (see Experimental). Nevertheless, averaging over a large number of individual measurements on three independent samples at nine different locations values of d = 72 ± 12 nm before rinsing and d = 66 ± 13 nm after rinsing were determined. Obviously, a slight drop of thickness by 9% after rinsing was found, which confirms the FTIR data on wet-adhesiveness for T = 25 °C in Table 4.

#### 3.2.3. Thermal Characterization of the Coating

**Figure 5:**
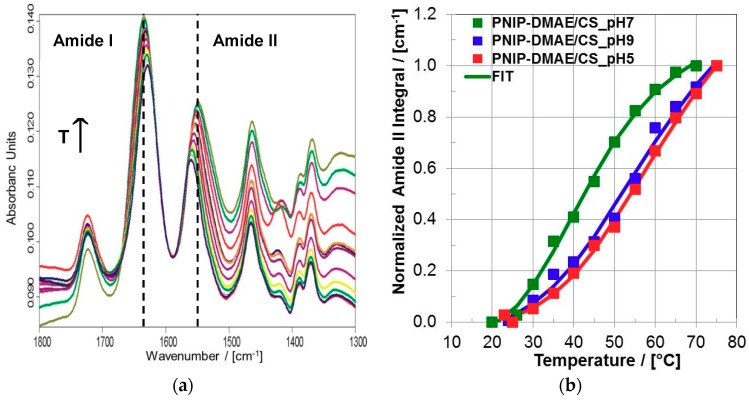
(**a**) Typical situ ATR-FTIR spectra on casted films of PNIPAM-DMAEMA/CS-1.2 in contact to HEPES buffer (pH = 9.0) for varying temperatures T (24 °C (dark blue, lowest Amide II intensity), 30, 35, 40, 45, 50, 55, 60, 65, 70, 75 °C (olive, highest Amide II intensity). Broken lines give the wavenumber positions of Amide I and Amide II band. (**b**) Typical plots of the diagnostic Amide II integrals in ATR-FTIR spectra of PNIPAM-DMAEMA/CS coatings in contact to aqueous media with pH = 9.0, 7.0 and 5.0 versus temperature T. Full Circles are related to experimental data points, full lines to fits based on Equation (1).

Since in the previous section PNIPAM-DMAEMA/CS coatings were proven to be adhesively stable in contact to water, they could be checked for their thermal response. Unlike PNIPAM-DMAEMA/CS dispersions, whose thermal properties were checked by turbidity measurements, thermal properties of PNIPAM-DMAEMA/CS coatings at aqueous media have to be checked by another technique such as in-situ ATR-FTIR spectroscopy. In Figure 5a typical ATR-FTIR spectra of the PNIPAM-DMAEMA coating are shown, which was in contact with the HEPES buffer at various temperatures from T = 20 °C to T = 75 °C.

The diagnostic IR bands have been introduced above. Both diagnostic wavenumber shifts and intensity increases of the Amide I and Amide II band due to PNIPAM-DMAEMA copolymer were obtained. Generally, processes like swelling and deswelling (e.g., solvent uptake and loss) of polymer films or hydrogels monitored by ATR-FTIR spectroscopy result in the respective decrease and increase of polymer related IR bands, since the polymer segment concentration is decreased or increased like it is shown therein [16]. Since such processes are considered to be closely related to the phase transition of PNIPAM based systems, such spectral changes were used as a measure of volume phase transition progress in the following. In the Figure 5b the integrals of the diagnostic Amide II band were plotted versus temperature for three different pH values. Significantly, the intensity courses versus T show sigmoidal curves, which are typical for phase transitions, like they have been obtained for PEC dispersions by turbidity (see Section 3.1.1). Similar to those, the FTIR related data were fitted by the sigmoidal function according to Equation (2). In Table 5, the parameters VPTT and cooperativity exponent P are listed.

Generally, the VPTT of PNIPAM-DMAEMA/CS coatings ranged from around 42 to 65 °C, which is again on average significantly higher compared to the VPTT of pure PNIPAM in solution [2] and considerably in the range of PNIPAM-DMAEMA/CS dispersions.

In detail, for the physiological pH = 7.0 values of VPTT = 41.8 °C and P = 5.8 were found by FTIR analysis for PNIPAM-DMAEMA/CS-1.2 coatings, which are comparable to the values of VPTT = 47.1 °C and of P = 6.2 found by turbidity analysis for PNIPAM-DMAEMA/CS-1.2 dispersions. For the lower pH = 5.0 as well as the higher pH = 9.0 higher values of VPTT = 64.1 and 61.3 °C and lower values of P around 4 were registered. These high VPTT values at these unphysiological pH values are of course rather unfeasible for biomedical applications, whereas the VPTT around 42 °C for pH = 7.0 is promising, as will be outlined below. The high VPTT (64.1 °C) of PNIPAM-DMAEMA/CS coatings for pH = 5.0, which is similar to that for PNIPAM-DMAEMA solutions at pH = 5.0 (59.6 °C, Table 2), can be explained straightforward by the protonation and higher charge density of DMAEMA units, which causes a higher hydration and thus retardation of volume phase transition. However, the high VPTT (61.3 °C) at pH = 9.0 for the coating, which is different to that for the pure PNIPAM-DMAEMA solution at pH = 9.0 (35.3 °C), is surprising on a first glance, since on the one hand DMAEMA units are expected to be uncharged at basic pH values and therefore the NIPAM units of the PEC should be less hydrated and the volume phase transition should set in earlier. However, taking into account that at pH = 9.0 upon discharging the DMAEMA units, the anionic units (sulfate groups) of CS are no longer intrinsically ion paired with the DMAEMA units (amino groups) and are thus better hydrated, the higher VPTT can be rationalized.

### 3.3. Thermoaddressable Delivery of Bone Therapeutic Proteins

Finally, the introduced PNIPAM-DMAEMA/CS coatings were applied for thermoresponsive delivery of the model protein papain (PAP) and bone morphogenetic protein (BMP-2) used for the therapy of acute bone defects associated with systemic bone diseases like osteoporosis or multiple myeloma (see Introduction). In principle no or only low elution of the protein after first contact to release medium at T = 37 °C and the triggerable onset followed by the sustained elution of the protein is aimed at, so that in future applications the surgeon can exactly define when the protein drug should start to elute from a given modified implant in the periprosthetic space. BMP-2 like PAP with isoelectric points IEP = 8.5 are basic proteins acquiring positive net charge in release media at pH = 7.0 and are thus expected to bind at the negatively charged excess CS component of PNIPAM-DMAEMA-1.2 coatings due to electrostatic attraction. More precisely, the driving force for the protein/CS interaction during uptake might be attributed to counterion evaporation, which was earlier claimed by Ballauff [17]. Whereas, release of proteins at elevated temperatures might be attributed to the loosening of ion pairs between protein and CS, which is induced by conformational changes of PNIPAM-DMAEMA also impacting the CS component.

#### 3.3.1. Loading and Elution Profiles of Functional Proteins

##### Papain

**Figure 6:**
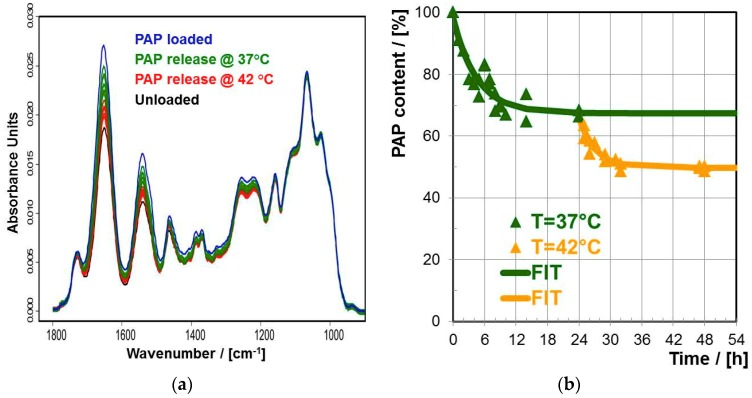
(**a**) Typical TRANS-FTIR spectra of PNIPAM-DMAEMA/CS-1.2 coatings before loading (black), after loading with PAP for 12h (blue) and after releasing PAP upon rinsing in HEPES buffer for 24 h at T = 37 °C (green), at T = 42 °C (red). (**b**) Release kinetics of papain (PAP) out of PAP loaded PNIPAM-DMAEMA/CS (PEC-1.2, 0.01 M) coatings at Ge for T = 37 and 42 °C into HEPES buffer based on TRANS-FTIR data given typically in Figure 6a.

In the following results on loading and releasing papain (PAP) at PNIPAM-DMAEMA/CS-1.2 coatings are shown. Concerning loading, Ge substrates functionalized by PNIPAM-DMAEMA/CS-1.2 coatings were immersed in 0.001 M PAP solutions at pH = 7.0 for 12h as it is described in the Experimental Section. In the Figure 6a FTIR spectra of PNIPAM-DMAEMA/CS-1.2 coatings are shown in the initial unloaded state, the PAP loaded state and the states after 24 h of PAP release at T = 37 and 42 °C immersed in HEPES buffer (pH = 7.0).

Ex-situ-TRANS FTIR spectroscopy was applied on samples in the dry state as introduced earlier [1]. Significantly, the intensity of the Amide II band at 1540 cm^−1^, which is indicative for protein uptake, increased from the unloaded state (bottom, black) to a maximum in the loaded state (top, blue) and further decreased again in the release regimes at T = 37 °C (green) and 42 °C (red), respectively. Note that during the whole experiment the band at 1065 cm^−1^ assigned to the ν(C–O) of ether and hydroxyl groups of CS kept constant, meaning that the PNIPAM-DMAEMA/CS coating kept adhesive (see above). In the Figure 6b the respective release kinetics of PAP from PNIPAM-DMAEMA/CS-1.2 coatings at T = 37 °C followed by 42 °C rationalized by the kinetic course of actual PAP content versus time is given, whose quantification was described in the Experimental Section. Typical release parameters can be obtained from these courses, like initial burst (IB) defined as the difference between the content after zero (100%) and after 1 h as well as residual content (RC) defined as protein content after 24 h, which are given in the Table 6. At T = 37 °C a low initial burst (IB = 9% after 1 h) followed by a slow liberation of PAP was obtained and after 24 h there was a constant relative PAP amount of 66% in the PEC coating. However, applying a temperature increase by 5 to 42 °C there was a further release of PAP starting with a low second burst and after another 24 h there was a reduced relative PAP amount of 50%. Conclusively, the model protein PAP could be released from thermoresponsive PEC coatings in two distinct steps by applying external heat, which is relevant for the defined release of therapeutic proteins with respect to time and location.

##### Bone Morphogenetic Protein (BMP-2)

**Figure 7:**
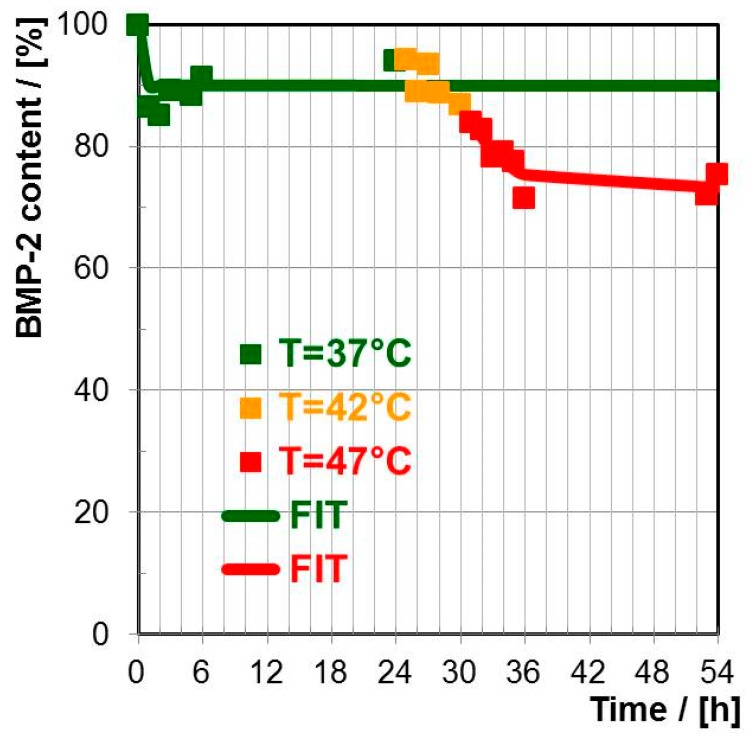
Release kinetics of BMP-2 out of BMP-2 loaded PNIPAM-DMAEMA/CS (PEC-1.2, 0.01 M) coatings at Ge for T = 37, 42 and 47 °C into HEPES buffer based on TRANS-FTIR data.

Analogously to the well available PAP, the PNIPAM-DMAEMA/CS-1.2 coatings on Ge substrate were loaded with the less available yet more relevant (bone healing) BMP-2 from 0.001 M protein solution. In the Figure 7 the BMP-2 release from thermoresponsive PNIPAM-DMAEMA/CS-1.2 coatings is shown at 37, 42 and 47 °C in HEPES buffer (pH = 7.0). As it is summarized in Table 6 at T = 37 °C a low initial burst (IB = 10% after 1 h) to 90% occurred and in the following no further BMP-2 was eluted until 24 h. Unlike for PAP the temperature increase to T = 42 °C for another six hours did not have a significant effect on a further release of BMP-2. Yet at T = 47 °C there was a further secondary release of BMP-2 with a “secondary initial burst” of 3% after one hour followed by a slow elution period, which leveled off at some 74% after another 24 h. Conclusively, BMP-2 could also be released in two distinct steps from thermoresponsive PNIPAM-DMAEMA/CS coatings by varying the temperature like it was shown for PAP. However, 47 °C was needed for the secondary release of BMP-2, which could be critical for the conservation of BMP-2 biological activity.

**Figure 8:**
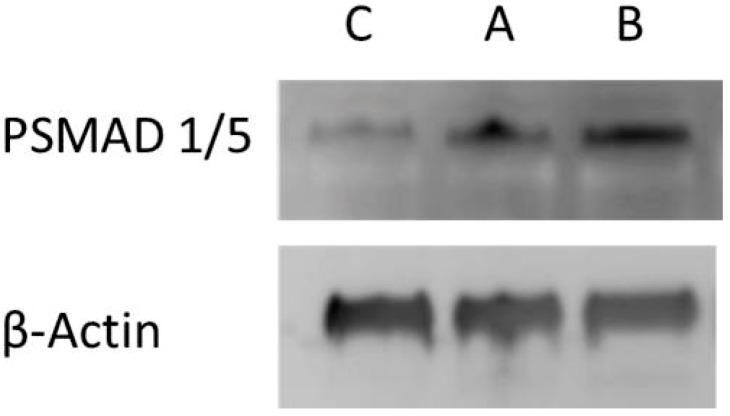
Biological activity of BMP-2 released from PNIPAM-DMAEMA coatings for 24 h at T = 37 °C (sample A) and T = 47 °C (sample B). The activation of SMAD mediated pathway in mouse C2C12 myoblasts stimulated by BMP-2 is shown by phosphorylation of SMAD 1/5 by Western Blotting on native and released BMP-2. For negative control an equal amount of buffer without BMP-2 was used (sample C).

Therefore, biological activity tests were performed for BMP-2 having been released after 24 h at T = 37 °C compared to T = 47 °C. C2C12 myoblasts were seeded in 6 well plates and incubated with the sample solutions containing released BMP-2 for T = 37 and 47 °C as well as cultured in absence of BMP-2 for negative control. Cells were lysed after 1 h and the bioactivity of BMP-2 was validated by BMP-mediated SMAD-dependent pathway signaling using western blotting as it was described [5]. SMADs are intracellular proteins, which transduce signals of extracellular transforming growth factors (TGF-b family) to cell nuclei activating transcription of genes involved in cell development [18]. SMAD 1/5 phosphorylation was detected relative to beta-actin. Figure 8 shows samples containing released BMP-2 induced SMAD 1/5 phosphorylation in C2C12 cells. In detail the band corresponding to released BMP-2 at 47 °C was stronger compared to the band for released BMP-2 at 37 °C and only a faint band was detected for buffer without BMP-2. Thus, the results show that both native and released BMP-2 samples were biologically active and indicate an increased BMP-2 signaling in cells cultured in medium with BMP-2 released from samples at higher temperature (47 °C), which contained a higher BMP-2 amount due to temperature controlled release enhancement.

#### 3.3.2. Thermorsponsive Release Mechanism and Physiological Relevance

**Figure 9:**
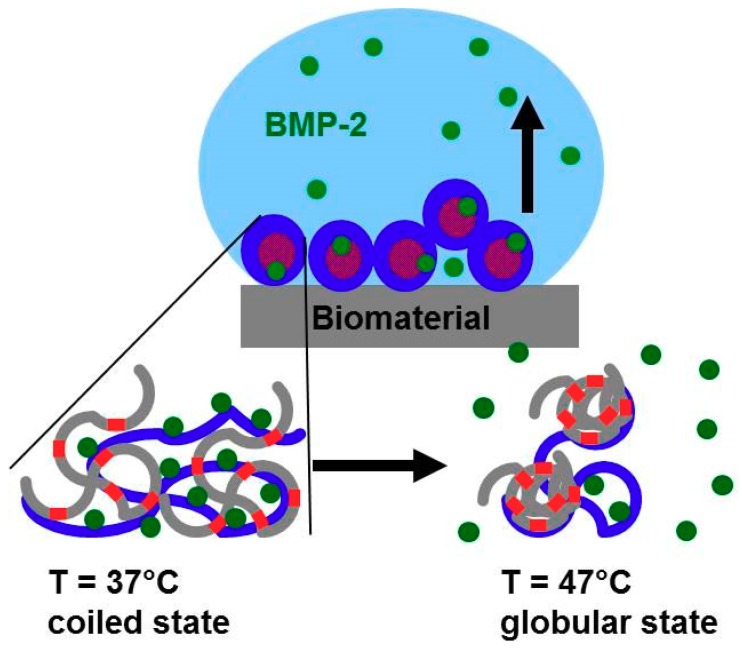
Scheme on the temperature dependent volume phase transition (VPT) and associated BMP-2 release scenario within PNIPAM-DMAEMA/CS complex coatings (blue and red color denote negative (CS) and positively (PDMAEMA) charged polymer units, respectively, grey color represents uncharged (PNIPAM) units and green dots the cationic proteins (BMP-2, PAP).

In the last section it could be shown, that PAP and BMP-2 release from PNIPAM-DMAEMA/CS coatings increases with increasing temperature. Intuitively, this was not expected in the first order, since PNIPAM containing systems are known to shrink and de-swell by the volume phase transition (VPT) above a certain VPTT around 34 °C and therefore the retention of a drug or a protein should be increased and slower release should occur upon respective temperature elevation. However, since both proteins PAP and BMP-2 are positively charged (IEP around 8.5) at the applied pH = 7.0, they are assumed to be bound rather to the anionic excess CS component of the negatively charged PNIPAM-DMAEMA/CS complex (n−/n+ = 1.2) than to the cationic deficit PNIPAM-DMAEMA component. Hence, as it is shown in the scheme of Figure 9, the VPT of NIPAM units in the random copolymer PNIPAM-DMAEMA is not likely to act directly on cationic protein binding via conformation change of cationic DMAEMA units but rather indirectly via induced conformation change of complexed anionic CS units.

This indirect perturbation of CS conformation changes modalities and degree of ion pairing between CS, DMAEMA and charged protein units (residues), which finally results in a partial protein release. Given the VPTT values of around 42 °C at the relevant pH = 7.0 (see Table 4) for PNIPAM/DMAEMA/CS coatings and the broad VPT (Figure 5b), release of the proteins (PAP, BMP-2) might set in even “earlier” at T < 42 °C.

Although this thermo-switchable protein release behavior is appealing for clinical applications, temperatures above T = 40 °C might be seen critical due to e.g., local thermal necrosis and protein denaturation. However, in bone surgery poly(methylmethacrylate) (PMMA) cements are frequently used, where a local and temporary heat up to T ≈ 70 °C upon polymerization of MMA is commonly achieved [19], which obviously is tolerable. Nevertheless, additional efforts should be followed to create confined local heat not only by external heating but also by external magnetic field variation like it is known for super paramagnetic iron oxide nanoparticles (SPION) [20,21], which are able to cause local hyperthermia.

## 4. Conclusions

Mixing the thermoresponsive cationic statistical copolyelectrolyte PNIPAM-DMAEMA with the anionic polysaccharide CS resulted in dispersed polyelectrolyte complex (PEC) particles with hydrodynamic radii in the range R_H_ = 170–288 nm at T = 25 °C. These PNIPAM-DMAEMA/CS-1.2 PEC particles show a significant temperature effect. Increasing the temperature to T = 60 °C PNIPAM-DMAEMA/CS particles shrank by factors of around 2–4 due to the known volume phase transition (VPT) associated with the coil/global conformation change of PNIPAM systems. Turbidity measurements on PNIPAM-DMAEMA solutions and PNIPAM-DMAEMA/CS dispersions at pH = 7.0 as a function of temperature revealed elevated VPT temperatures (VPTT) of ≈56 °C and ≈47 °C, respectively, in comparison to the known VPTT ≈ 34 °C of pure PNIPAM solutions at pH = 7.0. Casting and drying thermoresponsive PNIPAM-DMAEMA/CS PEC-1.2 dispersions at the Ge model substrate revealed thin coatings, which are adhesively stable in contact to various aqueous solutions. PNIPAM-DMAEMA/CS coatings showed a rather broad VPT, which could be evidenced by in-situ ATR-FTIR spectroscopy based on diagnostic shifts and intensity increases of the Amide I and Amide II band. VPTT values of around 42 °C were determined for pH = 7.0. Both at pH = 5.0 and pH = 9.0 higher VPTT values around 60 °C were found. While the high VPTT of the PNIPAM-DMAEMA/CS coating for pH = 4 might be caused by excessive cationic charge of the DMAEMA units, the high VPTT for pH = 9.0 might be caused by excessive uncomplexed CS units due to neutralization of DMAEMA units both resulting in higher hydration. PNIPAM-DMAEMA/CS-1.2 coatings could be loaded with the proteins papain (PAP) and bone morphogenetic protein (BMP-2) by immersing in a 0.001M protein solution at pH = 7.0 for periods >12 h. PAP and BMP-2 showed temperature dependent release behavior out of PNIPAM-DMAEMA/CS-1.2 coatings into HEPES buffer (pH = 7.0), where at T = 37 °C around 30% PAP and 10% BMP-2 were released after 24 h, which significantly increased to around 50% PAP and 30% BMP-2 upon temperature increase to T = 42 and 47 °C, respectively. This effect will be applied on biomaterials such as bone defect filling materials and osteosynthetic implants, while being aware of heat induced local physiological side effects.

## Figures and Tables

**Table 1:** Polymer (M_n_) and monomer (M) unit molecular weights and charge factors F of PNIPAM-DMAEMA, CS, PAP and BMP-2 at pH = 4.0 and pH = 7.0. (−) and (+) denote charge signs.

	M_n_/[g/mol]	M/[g/mol]	F (pH = 4.0)	F (pH = 7.0)
PNIPAM-DMAEMA	7.200	123 (average)	0.28–0.33 (+)	0.22–0.26 (+)
CS	100.000	213	0.36 (−)	0.39 (−)
PAP	24.000	100	0.07 *	0.04 *
BMP-2	30.000	100	--- **	--- **

* From [9], ** not determined (availability).

**Table 2:** VPTT and cooperativity (P) values found by fitting the turbidity courses of pure PNIPAM and PNIPAM-DMAEMA solutions (0.02 M) at pH = 7.0 and PNIPAM-DMAEMA/CS dispersions (0.01 M) at pH = 5.0, 7.0 and 9.0 versus temperature according to Equation (2).

Samples	VPTT	P
PNIPAM, pH = 7.0	34.1 ± 0.2 °C	34.5 ± 8.2
PNIPAM-DMAEMA, pH = 7.0	55.8 ± 0.3 °C	9.7 ± 0.4
PNIPAM-DMAEMA/CS-1.2, pH = 7.0	47.1 ± 0.5 °C	6.2 ± 0.3
PNIPAM-DMAEMA, pH = 5.0	59.6 ± 2.3 °C	4.2 ± 0.8
PNIPAM-DMAEMA, pH = 9.0	35.3 ± 0.3 °C	17.2 ± 2.2

**Table 3:** Hydrodynamic radii (R_H_) of PNIPAM-DMAEMA/CS complex dispersions (0.01 M) for n−/n+ = 0.9 and 1.2 at pH = 7.0 and 4.0 at T = 25 and 60 °C obtained by DLS.

PEC Dispersions	R_H_/[nm]	
	T = 25 °C	T = 60 °C
PNIPAM-DMAEMA/CS-0.9, pH = 7.0	171 ± 13	103 ± 38
PNIPAM-DMAEMA/CS-1.2, pH = 7.0	170 ± 45	76 ± 26
PNIPAM-DMAEMA/CS-0.9, pH = 4.0	--- *	--- *
PNIPAM-DMAEMA/CS-1.2, pH = 4.0	288 ± 55	74 ± 6

* samples instable (flocculation).

**Table 4:** Wet-adhesiveness W = A_AFTER_/A_BEFORE_ × 100% of PNIPAM-DMAEMA/CS-1.2 complex coatings for pH = 5.0, 7.0 and 9.0 and 0.1 M NaCl at T = 25 and 60 °C obtained by FTIR spectroscopy.

	W/[%]			
	T = 25 °C		T = 60 °C	
PEC Coatings	PNIPAM-DMAEMA	CS	PNIPAM-DMAEMA	CS
PNIPAM-DMAEMA/CS-1.2, pH = 5.0	90 ± 4	79 ± 3	90 ± 4	79 ± 2
PNIPAM-DMAEMA/CS-1.2, pH = 7.0	85 ± 6	79 ± 6	91 ± 7	82 ± 5
PNIPAM-DMAEMA/CS-1.2, pH = 9.0	81 ± 14	66 ± 13	83 ± 6	67 ± 8
PNIPAM-DMAEMA/CS-1.2, NaCl	78 ± 4	74 ± 3	93 ± 4	79 ± 3

**Table 5:** VPTT and P values found by fitting the courses of Amide II from ATR-FTIR spectra of PNIPAM-DMAEMA/CS-1.2 coatings versus temperature using Equation (2).

PEC Coating Samples	VPTT (FTIR)	P
PNIPAM-DMAEMA/CS-1.2, pH = 5.0	64.1 ± 3.5 °C	4.1 ± 0.4
PNIPAM-DMAEMA/CS-1.2, pH = 7.0	41.8 ± 1.6 °C	5.8 ± 0.4
PNIPAM-DMAEMA/CS-1.2, pH = 9.0	61.3 ± 7.0 °C	3.8 ± 0.9

**Table 6:** Initial burst (IB) after 1h and residual content (RC) after 24 h of the release kinetics of PAP and BMP-2 from PNIPAM-DMAEMA/CS-1.2 coatings at HEPES buffer in dependence of temperature.

Temperature	IB/[%]	RC/[%]
PAP, T = 37 °C	9%	66%
PAP, T = 42 °C	3%	50%
BMP-2, T = 37 °C	10%	90%
BMP-2, T = 42 °C	≈0%	87%
BMP-2, T = 47 °C	3%	74%
